# Variation in the rate of well-controlled status of chronic disease by income level in Korea: 2010 to 2015

**DOI:** 10.1097/MD.0000000000012059

**Published:** 2018-08-24

**Authors:** Kun Kug Choi, Seung Hyuk Kim, Kyung Don Yoo, Hyo Jin Kim, Ji In Park, Subin Hwang, Ho Jun Chin, Ho Suk Ku

**Affiliations:** a Department of Internal Medicine, Inje University Seoul Paik Hospital, Inje University School of Medicine; b Division of Nephrology, Department of Internal Medicine, Dongguk University Gyeongju Hospital, Gyeongju; c Department of Internal Medicine, Kangwon National University Hospital, Chuncheon; d Department of Internal Medicine, Seoul National University Bundang Hospital, Seongnam-si, Kyeonggi-do, Republic of Korea.

**Keywords:** chronic disease, chronic kidney disease, diabetes mellitus, education level, hypertension, income level, occupational status

## Abstract

Although it is known that the prevalence rates of chronic diseases depend on income level, annual changes of the control rate have not been evaluated. In this cross-sectional study, we analyzed the variation in rate of well-controlled status of chronic diseases based on the annual income level using data from national nutrition surveys conducted between 2010 and 2015.

Prevalence and controlled rate of hypertension, diabetes mellitus, and chronic kidney disease were analyzed in relation to annual income levels, using data from the Korea National Health and Nutrition Examination Survey (KNHANES), obtained from 2010 to 2015. We also analyzed the incidence of use of necessary medical care services and the reasons cited for not using these services.

The data of 28,759 persons were analyzed. The average age increased, and sex ratio remained unchanged over the study period. Although the prevalence rates of diabetes increased, that of increased glycated hemoglobin gradually decreased. A significant change has been shown recently on the prevalence rates of hypertension patients. The prevalence rates of chronic kidney disease stayed unchanged during the course of the study period. The incidence of controlled chronic disease status increased with the income level, and over time during the study, in the case of diabetes and chronic kidney disease. However, while controlled hypertension status rate increased from year to year, there was no trend of increase with increased income level. The incidence of participants not using hospital services declined with increasing income level, but the rate of economic causes being cited as reasons for not using hospital services increased over time and showed no change among income levels. Results of regression analysis of prevalence rates of chronic diseases by income level showed that lower income groups tended to have higher odds ratios for chronic diseases.

Our results suggest that the incidence rate of well-controlled chronic disease status remains low in lower income groups. These results imply that financial status may play an important role in the management of chronic diseases.

## 1 Introduction

In the Poverty Survey of 2015, the poverty rate of South Korea based on the minimum cost of living was reported to be nearly 12.5%.^[1]^ Of the 51 million population of the Republic of Korea in 2015, about 6.3 million were below the poverty line. Income, education, and occupational status, in addition to biological factors, are known to influence health status of individuals. The relationship between income and health is clear. People with low income cannot use medical facilities, and stress due to low income increases the risk for heart disease, cerebrovascular disease, and diabetes. When households were classified into 5 categories according to income, the mortality rate of those in the lowest income households was reported to be 1.56 times higher than the mortality rate of those in the highest income households, after correcting for various factors.^[2]^ Education affects health, since education enhances cognitive functioning, and cognitive factors affect health behavior and lifestyle. This implies that education can promote health through improved cognitive functioning. For instance, according to a report published in 1985, though income levels were low in Kerala, India, the reported infant mortality rate was also low due to a high literacy rate.^[3]^ Labor and working conditions are also important factors influencing health. Occupations with irregular working hours, in contrast to those with regular working hours, are known to adversely affect physical, mental, and psychological health.^[4]^ Accessibility to healthcare facilities^[4]^ and health insurance coverage may also affect an individual's health. In a study of mortality rates based on employment, the mortality rates in unemployed individuals was higher in the United States compared with that in Germany.^[5]^ Therefore, it is clear that if health services are inaccessible due to any reason, health status is adversely affected. It is known that chronic diseases are increasing in Korea at present, and the prevalence and degree of control of chronic diseases are affected by income level.^[6]^ However, the prevalence and the degree of control of chronic diseases according to income level have not been studied in the recent 6 years. In this study, using data from the National Health and Nutrition Survey conducted in Korea for a 6-year period (2010–2015), the degree of control of chronic diseases was examined in relation to income level, education level, and occupational status.

## 2 Materials and methods

### 2.1 Study design and settings

We analyzed data from the Korea National Health and Nutrition Survey obtained from 2010 to 2015 (6 years). The detailed design of the Korea National Health and Nutrition Examination Survey (KNHANES) is described in a previously published study.^[7]^ This survey was conducted by the Korean Ministry of Health and Welfare, and contained 3 sections: a health survey, health consultations, and a nutrition survey. The health questionnaire was used to survey income levels and the incidence of hypertension and diabetes. Health consultations were used to obtain data on blood pressure (BP), weight, waist circumference, body mass index (BMI), and levels of glycated hemoglobin (HbA1c), total cholesterol, high density lipoprotein, and serum creatinine. The survey data included information regarding the use of the necessary health care services, job type, and educational level. We divided the participants into 4 groups (low-, low-to-mid, mid-to-high, and high-income groups), according to the monthly average income in the household equivalence scale per year. The income was calculated as follows: household income/√number of household members. The income threshold groups were determined based on the quartiles of monthly average income on the household equivalence scale.

### 2.2 Definitions

Hypertension was defined as taking antihypertensive medication, a systolic blood pressure (SBP) of ≥140 mm Hg, or a diastolic blood pressure (DBP) of ≥90 mm Hg. Diabetes mellitus was defined as a fasting plasma glucose level of ≥126 mg/dL or taking anti-diabetic medication. Chronic kidney disease (CKD) was defined as an estimated glomerular filtration rate (eGFR) of <60 mL/(min·1.73 m^2^), according to the definition in the Modification of Diet in Renal Disease (MDRD) study.^[8]^ Estimated GFR was calculated by using the 4-variable MDRD formula as follows: eGFR (mL/min·1.73 m^2^) = 175 × serum creatinine level (mg/dL)^−1.154^ × age^−0.203^ × 0.742 (if female).^[8]^ The definition of the metabolic syndrome was based on the diagnosis category^[9]^ for the presence of ≥3 of the following: abdominal obesity of >90 cm in men or >80 cm in women; triglyceride level of ≥150 mg/dL or taking relevant medication; high-density lipoprotein cholesterol level of <40 mg/dL in men or <50 mg/dL in women, or taking relevant medication; SBP ≥130 mm Hg and/or DBP ≥85 mm Hg or taking relevant medication; fasting plasma glucose level of ≥100 mg/dL or taking relevant medication. Thresholds for abdominal obesity were defined based on those published for the Asian population.^[10]^

In the patients with hypertension, well-controlled BP was defined as BP controlled to <140/90 mm Hg according to the Joint National Committee (JNC) 8th Directive. In patients with diabetes mellitus (DM), controlled blood glucose level was defined as a HbA1c level of <7%. Well-controlled CKD was defined as negative proteinuria in a dip-stick test.

Educational level was expressed as a number as follows: completed up to elementary school graduation; completed middle school; completed high school; and obtained a college level degree or higher.

A total of 48,482 survey respondents were investigated, and those who did not report spot urine sodium level and economic status were excluded from the study. At result, 28,759 participants were included in the study.

The KNHANES was approved by the institutional review board of the Korea Centers for Disease Control (KCDC) and Prevention, and all the participants provided written informed consent. The study protocol conformed to the ethics guidelines of the 1975 Declaration of Helsinki and was approved by the institutional review board of the KCDC (for 2010 to 2012, No. 2010-02CON-21-C, 2011-02CON-06-C, 2012-01EXP-01-2C, for 2013–2015, No 2013-07CON-03-4C, 2013-12EXP-03-5C, 2015-01-02-6C).

### 2.3 Statistics

For continuous variables, to evaluate the subjects’ characteristics, a descriptive analysis was performed. Categorical variables were analyzed by using the chi-square test to determine the correlation between different variables. We divided the subjects into 4 groups according to income level to allow for comparison of variables. To determine differences in characteristics between the groups, we used the analysis of variance method. We performed regression analysis of the incidence of chronic diseases, and adjusted for confounding factors such as age, BMI, SBP, DBP, HbA1c level, and serum creatinine level. Significance was defined as a *P* value <.05. R version 3.3.1. (R Foundation for Statistical Computing, Vienna, Austria) was used for performing the statistical analyses.

## 3 Results

### 3.1 Baseline characteristics

The mean age of the participants increased from 2010 to 2012, and decreased in 2013, subsequently increasing again until 2015. The sex ratio was high for women in the 6-year study period between 2010 and 2015. The year-wise proportion of participants in the upper income group fell from 2010 to 2011, but thereafter remained fairly constant till 2015. The proportion of participants in the upper middle income group did not show significant differences from 2010 to 2014, but declined in 2015. The lower middle income group increased from 2010 to 2011, then decreased from 2012 to 2013, and subsequently increased from 2014 to 2015. The proportion of participants in the lower income group showed an upward trend until 2013, but decreased from 2014 onwards. The number of current smokers remained fairly constant from 2010 to 2014, but fell significantly in 2015. The smoking rate was about 20% during the 6-year study period. The prevalence of hypertension averaged over 30%, declining to 19.9% in 2014 and rising again to 32.6% in 2015. The prevalence of diabetes was about 10%, showing a steady increase over the 6-year period. The incidence of GRF <60 increased to about 3.0% by 2012 and subsequently remained constant until 2015. The incidence of the metabolic syndrome was 25% in 2010, but decreased to 10.2% in 2014 and increased to 19.0% in 2015. The highest value of HbA1c, at 7.27%, was observed in 2010, which decreased to an average value of 5.7% for the period from 2011 to 2015. During the 6-year study period, the percentage of elementary school graduates decreased, and the percentage of college level graduates increased. The proportion of temporary workers showed an increasing trend through the study period, while there was no identifiable trend in the change in proportion of regular and daily workers (Table 1).

**Table 1:**
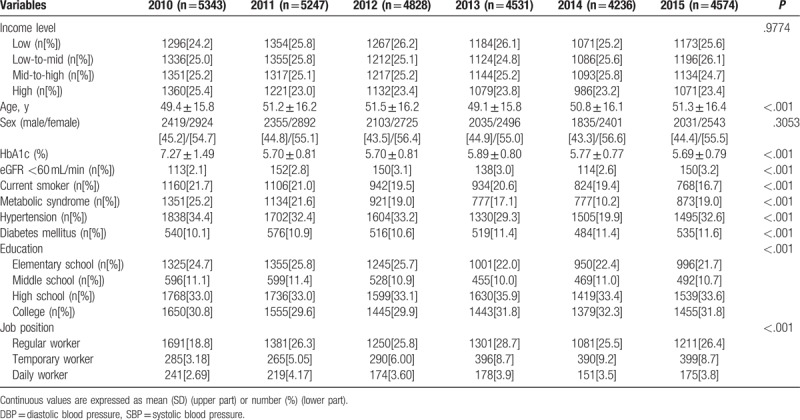
Baseline characteristics of participants (n = 48,832,940; weighted).

### 3.2 The prevalence of maintaining the recommended blood pressure level in hypertension, glucose level in diabetes mellitus, and proteinuria in chronic kidney disease

**Figure 1:**
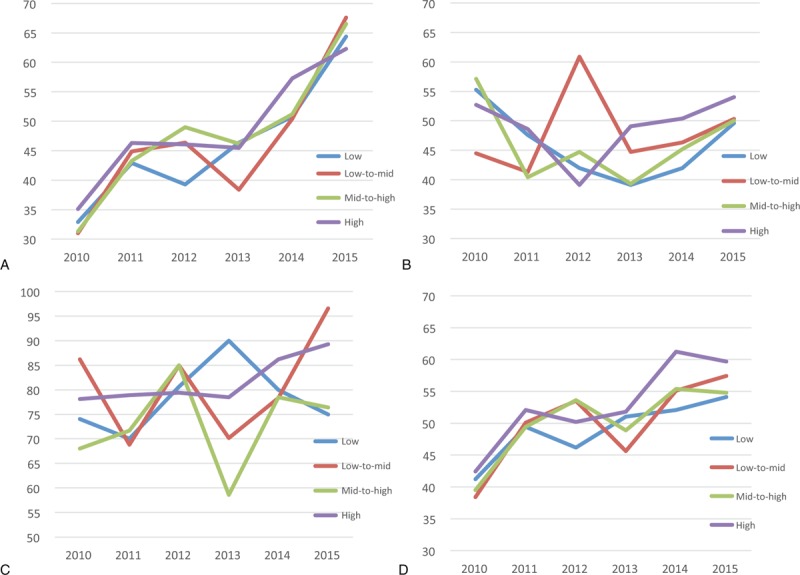
(A) Data on incidence of maintaining recommended blood pressure in patients with hypertension graphed according to income level and year. (B) Data on incidence of maintaining the recommended glycated hemoglobin levels in patients with diabetes mellitus, graphed according to income level and year. (C) Data on incidence of maintaining negative proteinuria in patients with chronic kidney disease, graphed according to income level and year. (D) Data on incidence of well-controlled chronic disease (hypertension, diabetes mellitus, and chronic kidney disease) graphed as per income level and year.

The proportion of participants with well-controlled hypertension increased during the 6-year period and rose to 65.2% in 2015. The rate of well-controlled hypertension was found to be high in the 6-year period in those with higher incomes (Fig. 1A). The proportion of diabetic participants with HbA1c <7.0% increased over the 6-year study period and reached 51.0% in 2015. The proportion of patients with HbA1c <7.0% was higher in higher income group (Fig. 1B). The percentage of participants with CKD who were negative for proteinuria increased during the 6-year period (reached 84.6% by 2015), and was found to be higher in the higher income groups (Fig. 1C). The degree of chronic disease control increased steadily during the 6 years, and rose to 56.7% in 2015. The control rate also increased with the increase in income level, and higher income levels showed a higher degree of disease control (Fig. 1D) (Table 2).

**Table 2:**
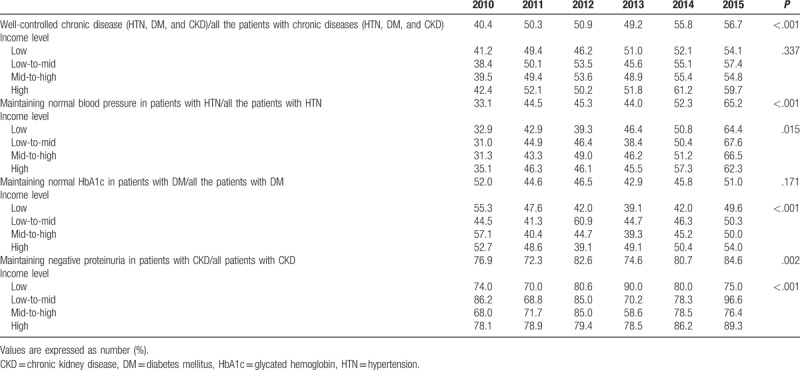
The prevalence of maintaining recommended blood pressure in hypertension, glucose level in diabetes mellitus, and proteinuria in chronic kidney disease (n = 48,832,940; weighted).

### 3.3 The prevalence of and reasons for not using the necessary medical services: survey results

The rate of failure to use essential health care decreased during the 6-year study period. However, 14.4% of participants were still observed to not use essential medical care in 2015, and this number increased with a decrease in the income level; therefore, the percentage of people who did not use essential medical care was higher in the lower income groups. Furthermore, those who did not access essential medical care and cited economic conditions as causative factors, more often belonged to the low income groups. Our data shows that from 2013 to 2015, about 40% of participants in the low income group cited economic reasons for not using essential medical care. About 2% of the participants cited difficulty in obtaining admission at a hospital as the reason for not using essential medical care. Traffic inconvenience was cited as the reason more often by the lower income groups and time constraints were cited as the reason more frequently in the higher income groups. The reason “The hospital was not open at the time when I could go” was cited as the reason more often by the higher income groups. The reason “Do not want to wait a long time in the hospital” was no difference in each income groups. The reason “the symptoms were not severe enough to go to the hospital” was cited more frequently as the income level increased (Table 3).

**Table 3:**
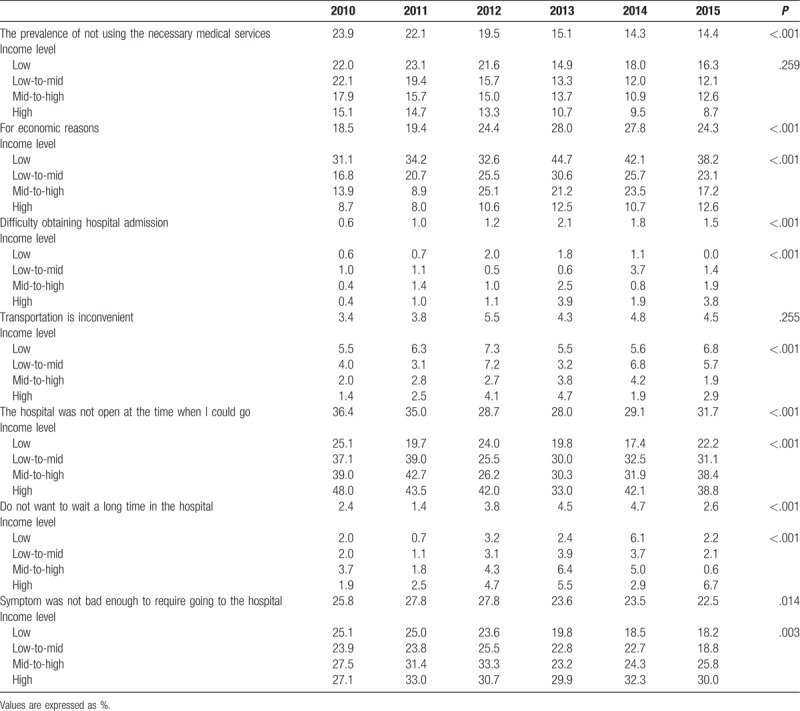
The prevalence of and the reasons cited for not using the necessary medical services: survey results (n = 48,832,940; weighted).

### 3.4 Results of the logistic regression analysis of prevalence of chronic diseases (hypertension, diabetes mellitus, and chronic kidney disease)

We analyzed the factors affecting the prevalence of chronic diseases, and found that age, BMI, SBP, DBP, low income level, and occupation as a regular worker were significant factors throughout the study period. Except for the year 2011, low income level was identified as a factor affecting the prevalence of chronic diseases during the rest of the study period (Table 4).

**Table 4:**
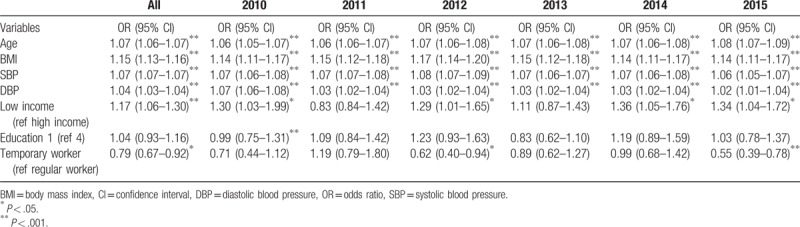
Results of the logistic regression analysis of prevalence of chronic diseases (hypertension, diabetes mellitus, and chronic kidney disease).

## 4 Discussion

According to the Ministry of Health and Welfare, the prevalence of chronic diseases such as hypertension, diabetes, and hyperlipidemia is increasing steadily in Korea.^[11]^ The prevalence of hypertension in the recent 6 years (2010–2015) is reported to be about 30.0%. Our data, in agreement with the above showed that the prevalence rate increased from 25.4% to 28.9% during the period from 2010 to 2015. The incidence of DM and CKD has also been reported be steadily increasing, reaching 11.3%^[11]^ and 16.5%,^[12]^ respectively in 2013. It is expected that the prevalence and burden of chronic diseases will continue to increase due to the increase in the elderly population.^[13]^ In our study, the prevalence of chronic diseases fluctuated annually. The average incidence of hypertension, DM, and CKD was 30%, 10%, and 3.2%, respectively.

Chronic disease incidence has also been reported to differ according to income level, and lower income levels have been reported to be associated with higher prevalence rates of hypertension, DM, and hyperlipidemia.^[6,14]^ While the incidence rates of chronic diseases have been well studied, the degree of control of these diseases is relatively unknown. Recent studies have shown that the controlled rate was 62.2% for hypertension and 22.1% for DM during 2010 to 2012.^[15]^ However, the degree of proteinuria control in patients with CKD is unknown. In our study, the overall rate of hypertension control increased through the study period, and in 2015, the appropriate blood pressure was maintained in 65.2% of the cases. In the case of DM, the trend in the degree of control varied through the study period, and in 2015, it was observed that 51.0% of participants were able to maintain the recommended blood glucose levels. Better control was observed in CKD than in hypertension and DM; proteinuria was not observed in an average of 84.6% of CKD cases in 2015. The degree of control of these chronic diseases is reported to depend not only on biological factors, but also on socioeconomic factors such as education, job status, and income levels.^[2,3]^ Among these factors, in this study, the degree of control varied significantly according to income level. The controlled status rate as determined by maintaining the recommended blood pressure in hypertensive participants, and maintaining glycemic control in diabetic participants decreased with a decrease in the income level throughout the 6-year study period. The incidence of proteinuria was higher in participants with CKD at lower income levels. The use of essential medical care is important for the management of chronic diseases, and in our study, an additional survey revealed that economic factors were cited by many respondents as the reason for not using essential medical care. The percentage of respondents citing this reason did not change throughout the 6-year study period.

The World Health Organization's (WHO) “Social Determinants of Health Committee” defined in 2008 the social determinants of health based on a comprehensive review.^[16]^ The Committee noted that the low level of health of the poor is due to the social disparities in health determinants and the significant national health inequalities arising from unequal distributions of power, income, goods, and services within the country; the committee has strongly recommended policy changes accordingly.^[16]^ In Korea, a poverty survey has revealed that 3.4 million people lived in poverty in 2010. If the population in the income level just above the poverty line is included (income at100–120% of the minimum cost of living), the total number of people at this level was 5.7 million in 2010.^[1]^ The results of our study suggest that treatment of chronic diseases in the poor is more difficult since economic constraints may prevent the use of essential medical facilities; thus, our study reiterates that economic reasons important social determinants of health. In other words, the social circumstance that leads to the circle of poverty → health → education → labor → poverty is further aggravated when welfare or social policy is inadequate. It can be termed as the “vicious cycle of health inequality.”

Risk factors for chronic illness including unhealthy diet, obesity, hyperglycemia, hypertension, and smoking are known to be related to poverty as well. Poverty leads to expose to the above mentioned risk factors of chronic diseases and additionally, poverty-related economic constraints prevent the necessary use of appropriate medical facilities.^[17]^ In our study, age, BMI, SBP, and DBP were identified as risk factors for the prevalence of chronic diseases. We observed a significant direct correlation between incidence of chronic disease and income level, though the association between chronic disease and education level was not clear.

This study has the following limitations. Financial instability, anxiety, and chronic stress due to social deprivation and low social status, which are the causes of health inequality caused by economic differences, are also known to cause cardiovascular and immune system deterioration. In this study, we did not evaluate the effect of these factors, and further investigations of the effect of psychological problems on chronic disease control are indicated. The prevalence of CKD was estimated to be relatively low, as determined by GFR data alone, and the degree of control of CKD was estimated to be high, based on proteinuria data alone. Hence, further parameters related to CKD need to be included in future analyses to confirm our results. In addition, in our study, the prevalence of chronic diseases was lower in temporary workers than that in regular workers, which is contrary to the generally accepted view that chronic diseases are more prevalent among temporary workers. Therefore, the results of this study need to be evaluated with the inclusion of other occupational factors such as working time and working style.

In summary, our results suggest that the gap in the chronic disease prevalence rates between high and low income levels has decreased. However, the rate of control of the incident chronic disease still remains low in lower income groups, implying that financial burden may play an essential role in the management of chronic disease.

## Author contributions

**Kun Kug Choi:** data analysis, manuscript writing.

**Seung Hyuk Kim:** data analysis, data cleansing.

**Kyung Don Yoo:** data analysis.

**Hyo Jin Kim:** data analysis.

**Ji In Park:** data analysis.

**Subin Hwang:** data analysis.

**Ho Jun Chin:** data analysis.

**Ho Suk Ku:** data manipulation, manuscript writing.

**Data curation:** Kun Kug Choi, Seung Hyuk Kim, Kyung Don Yoo, Hyo Jin Kim, Ji In Park, Su Bin Hwang, Ho Jun Chin, Ho Suk Ku.

**Writing – original draft:** Ho Suk Ku.

**Writing – review & editing:** Kun Kug Choi, Ho Suk Ku.
